# Supplementary material for: Changes in Children and Youth’s Mental Health Presentations during COVID-19: A Study of Primary Care Practices in Northern Ontario, Canada
Source: Int J Environ Res Public Health. 2023 Aug 17;20(16):6588. doi: 10.3390/ijerph20166588 (PMC10454016; doi:10.3390/ijerph20166588)
Supplement: Supplementary file 1 [file ijerph-20-06588-s001.zip › ijerph-2509238-supplementary.pdf]

Changes in Children and Youth's Mental Health Presentations During COVID-19: A Study of Primary Care Practices in Northern Ontario, Canada

Roya Daneshmand, PhD [1,2]*, Shreedhar Acharya, MPH[2], Barbara Zelek, MD[2], Michael Cotterill, MD[2], Brianne Wood, PhD[1,2]*

Supplemental Materials

Table S1. List of antidepressant/antianxiety prescriptions used for patients who diagnosed with anxiety or depression

Class	Generic	Brand Name
SSRI	citalopram	Celexa
	fluoxetine	Prozac
	sertraline	Zoloft
	escitalopram	Cipralex
	paroxetine	Paxil
	fluvoxamine	Luvox
SNRI	duloxetine	Cymbalta
	venlafaxine	Effexor
	desvenlafaxine	Pristiq
	levomilnacipran	Fetzima
Other	bupropion	Wellbutrin
	mirtazapine	Remeron
	vortioxetine	Trintellix
	vilazodone	Viibryd
	agomelatine	Valdoxan
	moclobemide	Mannerix
	lorazepam	
	clonazepam	
	alprazolam	
	diazepam	
	oxazepam	

Table S2. NORTHH patients' cohort characteristics

Age, year	Male, N (%)	Female, N (%)
< 10	1034 (8.8)	1003 (8.6)
10-25	2048 (17.4)	1926 (16.6)
26-50	3228 (27.4)	3300 (28.4)
51-65	2832 (24.1)	2695 (23.2)
65 <	2624 (22.3)	2685 (23.2)

Table S3. Comparing the predicted rate of primary care visit in children and youth between pre- and during the COVID-19 pandemic

	Predicted Rate of Primary Care Visit per 10000 person-month in Pre-Pandemic	Predicted Rate of Primary Care Visit per 10000 person-month- During Pandemic	Relative Change in Predicted Rate of primary care visit

Changes in Children and Youth's Mental Health Presentations During COVID-19: A Study of Primary Care Practices in Northern Ontario, Canada

Roya Daneshmand, PhD [1,2]*, Shreedhar Acharya, MPH[2], Barbara Zelek, MD[2], Michael Cotterill, MD[2], Brianne Wood, PhD[1,2]*

	(June 1, 2018-February 28, 2020)			(April 1, 2020-December 31, 2021)					
	**Total**	**Male**	**Female**	**Total**	**Male**	**Female**	**Total**	**Male**	**Female**
**Total**	72.3	217.3	194.4	117.8	325.0	319.9	62.9	49.6	64.6
**Age, years**									
10-14	193.2	238.2	139.5	281.8	97.1	431.2	45.9	-59.2*	209.0
15-19	160.4	230.1	144.8	442.0	402.8	425.0	175.6	75.0	193.4
20-25	224.7	204.1	238.3	267.4	335.7	226.4	19.0	64.5	-5.0 *

* Non-significant

Table S4. Comparing the predicted rate of antidepressant/antianxiety prescriptions in children and youth between pre- and during the COVID-19 pandemic

	Predicted Rate of Prescription per 10000 person-month in Pre-Pandemic (June 1, 2018-February 28, 2020)			Predicted Rate of Prescription per 10000 person-month-During Pandemic (April 1, 2020-December 31, 2021)			Relative Change in Predicted Rate of prescription		
	**Total**	**Male**	**Female**	**Total**	**Male**	**Female**	**Total**	**Male**	**Female**
**Total**	222.4	232.7	211.0	296.9	256.3	317.4	33.5	10.1	50.4
**Age, years**									
10-14	70.5	65.1	50.2	774.6	843.0	630.6	998.9	1194.2	1156.5
15-19	192.6	219.6	189.3	335.3	325.7	334.2	74.1	48.3	76.5
20-25	254.2	248.5	256.8	225.3	205.4	233.5	-11.3	-17.3	-9.1
